# Antagonizing Sec62 function in intracellular Ca^2+^ homeostasis represents a novel therapeutic strategy for head and neck cancer

**DOI:** 10.3389/fphys.2022.880004

**Published:** 2022-08-15

**Authors:** Sandrina Körner, Tillman Pick, Florian Bochen, Silke Wemmert, Christina Körbel, Michael D. Menger, Adolfo Cavalié, Jan-Philipp Kühn, Bernhard Schick, Maximilian Linxweiler

**Affiliations:** ^1^ Department of Otorhinolaryngology, Head and Neck Surgery, Saarland University Medical Center, Homburg, Germany; ^2^ Experimental and Clinical Pharmacology and Toxicology, Pre-Clinical Center for Molecular Signalling (PSMZ), Saarland University, Homburg, Germany; ^3^ Institute for Clinical and Experimental Surgery, Saarland University, Homburg, Germany

**Keywords:** Sec62, head and neck cancer, CRISPR/ Cas9, lymphogenic metastasis, xenograft model, trifluoperazine, thapsigargin

## Abstract

Various cancer types including head and neck squamous cell carcinomas (HNSCC) show a frequent amplification of chromosomal region 3q26 that encodes, among others, for the *SEC62* gene. Located in the ER membrane, this translocation protein is known to play a critical role as a potential driver oncogene in cancer development. High *SEC62* expression levels were observed in various cancer entities and were associated with a poor outcome and increased metastatic burden. Because of its intracellular localization the SEC62 protein is poorly accessible for therapeutic antibodies, therefore a functional *SEC62* knockdown represents the most promising mechanism of a potential antineoplastic targeted therapy. By stimulating the Ca^2+^ efflux from the ER lumen and thereby increasing cellular stress levels, a functional inhibition of SEC62 bears the potential to limit tumor growth and metastasis formation. In this study, two potential anti-metastatic and -proliferative agents that counteract SEC62 function were investigated in functional *in vitro* assays by utilizing an immortalized human hypopharyngeal cancer cell line as well as a newly established orthotopic murine *in vivo* model. Additionally, a CRISPR/Cas9 based *SEC62* knockout HNSCC cell line was generated and functionally characterized for its relevance in HNSCC cell proliferation and migration as well as sensitivity to SEC62 targeted therapy *in vitro*.

## Introduction

Head and neck squamous cell carcinomas (HNSCCs) belong to the eight most common cancers worldwide and account for more than 5% of all human malignancies (Bray et al., 2018). Beside chronic nicotine and alcohol consumption an infection of the oral/pharyngeal mucosa with high-risk human papillomaviruses was identified as a relevant risk factor for the development of an increasing portion of HNSCCs, which also constitutes a relevant prognostic factor and predictive biomarker for therapy response (Lewis et al., 2015; Lechner et al., 2019; Kühn et al., 2021). Compared to other human tumor entities HNSCC patients show a poor clinical outcome with a 5-year overall survival (OS) rate of 50–60% which has not markedly changed over the past decades (Fakhry et al., 2017; Horton et al., 2019). One reason for this unfavorable prognosis is the lack of efficient, disease-specific and targeted therapeutic strategies. Apart from the clinical approval of epidermal growth factor receptor (EGFR) antibody cetuximab and programmed cell death protein 1 (PD-1) antibodies nivolumab and pembrolizumab for recurrent and/or metastatic diseases, therapy concepts have not substantially changed over the past 15 years (Graham, et al., 2004; Ghanizada et al., 2019). In addition, the persisting delay of clinical diagnosis poses barriers to a successful curative treatment for the majority of HNSCC patients (Goy et al., 2009). Even if curation seems to be achieved in the initial treatment setting, more than half of the patients develop tumor recurrence in the first 5 years of follow-up (Koo et al., 2006; Haddad and Shin, 2008). Hence, a better understanding of molecular cell biology and the development of new approaches for targeted and efficient therapy are urgently needed in head and neck oncology.

**FIGURE 1:**
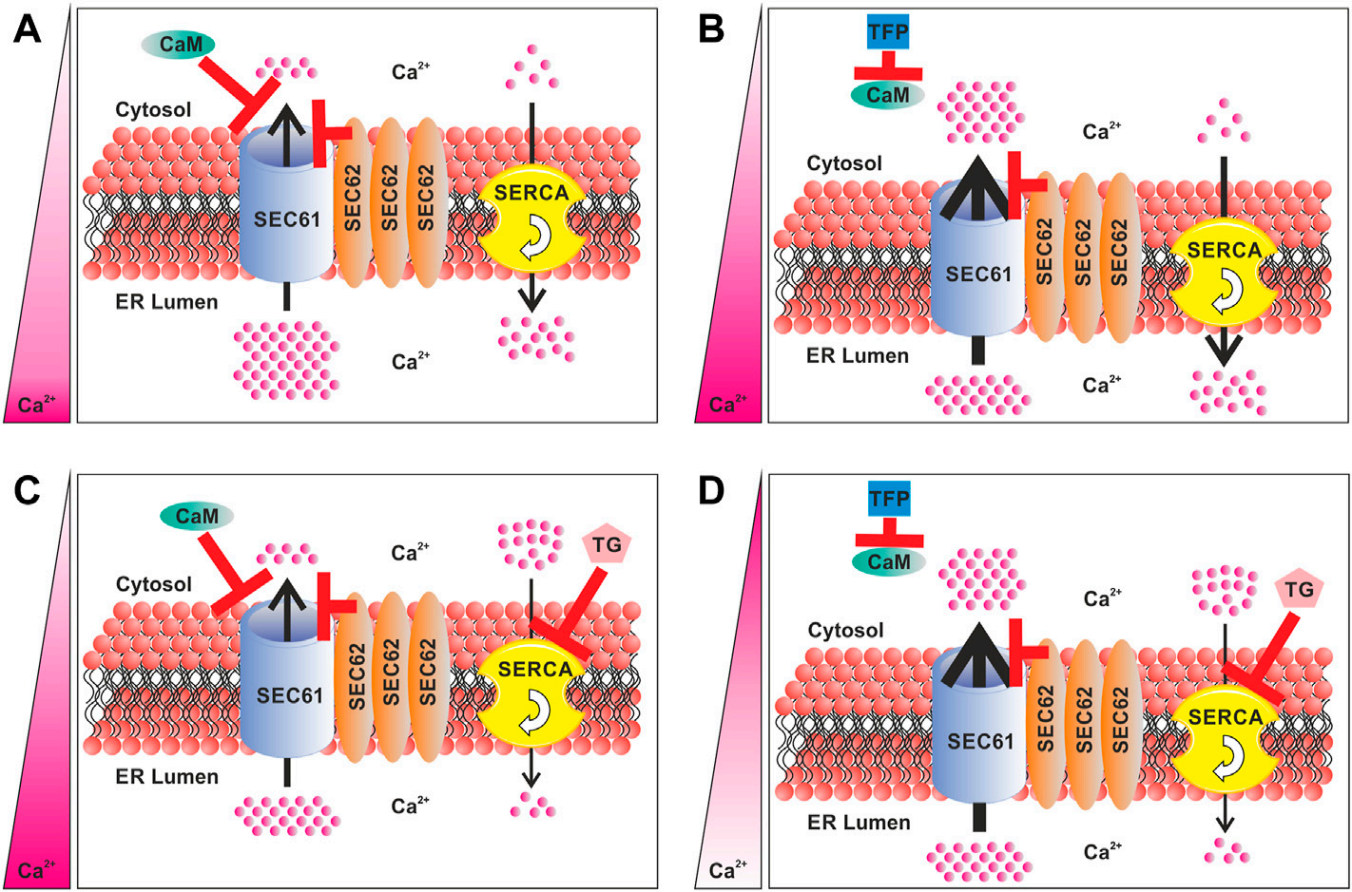
Components of the Ca^2+^ homeostasis regulation of the ER in *SEC62* overexpressing cancer cells. **(A)** Role of *SEC62* amplification in regulation of the Ca^2+^ efflux. **(B)** Differences in the Ca^2+^ efflux and resulting Ca^2+^ homeostasis under TFP treatment by the inhibition of CaM. **(C)** Differences in the Ca^2+^ efflux and resulting Ca^2+^ homeostasis under TG treatment by inhibition of the SERCA. **(D)** Differences in the Ca^2+^ efflux and resulting Ca^2+^ homeostasis under a consecutive treatment of TFP and TG by blocking CaM and through the inhibition of the SERCA. The red bars indicate an inhibition and/or channel closure. Thickness of the arrows indicates the strength of Ca^2+^efflux and the flow rate of the SERCA. The pink bar on the left indicates the Ca^2+^ balance between the ER and the cytosol. A dark pink color indicates a high Ca^2+^ level, whereas white color indicates a low Ca^2+^ level. CaM, calmodulin; TFP, trifluoperazine; TG, thapsigargin; SERCA, sarcoplasmic/endoplasmic reticulum Ca^2+^-ATPase.

To further uncover relevant molecular mechanisms of HNSCC carcinogenesis and tumor cell biology, several groups investigated genetic alterations in HNSCC tissue samples showing amplification of the long arm of chromosome 3 (3q) as highly characteristic for this tumor entity (Abou-Elhamd and Habib, 2008). Beside HNSCCs, also other human malignancies show a high incidence of 3q amplification including non-small cell lung cancer (NSCLC), cervical cancer and esophageal cancer (Chujo et al., 2002; Yen et al., 2005; Caraway et al., 2008). Encoded in chromosomal region 3q26.2 the *SEC62* oncogene was shown to be a relevant molecular target which is overexpressed in NSCLC, hepatocellular carcinoma, prostate cancer, cervical cancer, melanoma, and breast cancer (Jung et al., 2006; Weng et al., 2012; Hagerstrand et al., 2013; Linxweiler et al., 2016; Takacs et al., 2019; Casper et al., 2021; Müller et al., 2021; Sicking et al., 2021). *SEC62* overexpression was also found in 86% of HNSCC cases (Wemmert et al., 2016). Under physiological conditions the encoded SEC62 protein, located in the membrane of the endoplasmic reticulum (ER), is involved in intracellular protein translocation, the compensation of ER stress and the regulation of cellular calcium homeostasis (Lakkaraju et al., 2012; Lang et al., 2012; Linxweiler et al., 2013; Fumagalli et al., 2016; Linxweiler et al., 2017). Increased expression levels stimulate the migration and invasion potential as well as stress tolerance of cancer cells (Linxweiler et al., 2012, 2013, 2016; Bochen et al., 2017; Linxweiler, Schick and Zimmermann, 2017; Sicking et al., 2021). As a consequence, high *SEC62* expression levels were found to be associated with a higher incidence of lymphogenic and/or distant metastasis in HNSCC, NSCLC, melanoma, and breast cancer patients (Linxweiler et al., 2012; Bochen et al., 2017; Takacs et al., 2019; Müller et al., 2021). Additionally, high *SEC62* expression was associated with a significantly shorter overall survival in HNSCC (Wemmert et al., 2016; Bochen et al., 2017), breast cancer (Takacs et al., 2019), and NSCLC (Linxweiler et al., 2013), a significantly shorter progression-free survival (PFS) in melanoma (Müller et al., 2021) and HNSCC (Wemmert et al., 2016) as well as a shorter recurrence-free survival in hepatocellular carcinoma (Weng et al., 2012; Du et al., 2019). The migration and invasion stimulating effects of *SEC62* overexpression go along with a worse clinical outcome suggesting the SEC62 protein as a promising point-of-action for targeted therapeutic strategies. Indeed, a siRNA-mediated *SEC62* knock-down *in vitro* led to a highly effective inhibition of cancer cell migration in HNSCC, NSCLC, prostate cancer, and cervical cancer cells (Greiner et al., 2011; Linxweiler et al., 2012, 2016; Bochen et al., 2017). However, as the concept of siRNA-mediated *SEC62* knock-down cannot directly be transferred to an *in vivo* application due to delivery problems and severe adverse effects (Bonetta, 2009; Schmidt, 2011), alternative therapeutic strategies to counteract SEC62 function need to be found. In this context, calmodulin-antagonist Trifluoperazine (TFP) and sarcoplasmic/endoplasmic reticulum calcium ATPase (SERCA) inhibitor Thapsigargin (TG) were identified as small molecules that can mimic the molecular effects of a *SEC62* knock-down both on cellular calcium homeostasis, based on their interaction with the SEC61 channel and the intracellular calcium ATPase SERCA (Lang et al., 2012; Linxweiler et al., 2013) (Figure 1). So far, *in vitro* studies showed that TG and TFP can suppress the migration of various human cancer cells (Linxweiler et al., 2013). While this phenotype could be rescued by *SEC62* overexpression (Linxweiler et al., 2013), a *SEC62* knock-down sensitized tumor cells to TG- and/or TFP-induced ER-stress (Linxweiler et al., 2012) proving this concept of anti-metastatic and potentially anti-proliferative therapy. Correspondingly, a first *in vivo* study found a growth suppression of subcutaneously injected HNSCC cells in BALB/cAnNRj-Foxn1nu mice by TG and TFP (Körbel et al., 2018). However, to date no orthotopic mouse model was investigated which would allow analyses of orthotopic tumor growth and lymphogenic metastasis. Hence, more detailed and valid *in vivo* data are needed to facilitate a successful translation to clinical practice and the initiation of clinical studies.

Against this background, we established an orthotopic HNSCC murine xenograft model to investigate the potential anti-proliferative and anti-metastatic effects of TG and TFP *in vivo*. Additionally, a CRISPR/Cas9 based *SEC62* knockout HNSCC cell line was generated and functionally characterized for its relevance in cell proliferation, migration as well as sensitivity to a SEC62 targeted therapy *in vitro*.

## Materials and methods

### Human cell culture

For tumor cell preparation as well as for knockout-generation FaDu cells (DSMZ no. ACC 784) were cultured in Dulbecco’s Modified Eagle Medium (DMEM; Gibco Invitrogen, Karlsruhe, Germany) containing 10% fetal bovine serum (FBS; Sigma-Aldrich Chemie GmbH, Taufkirchen, Germany) and 1% penicillin/streptomycin (Sigma-Aldrich Chemie GmbH, Taufkirchen, Germany) in a humidified environment containing 5% CO_2_ at 37°C. Cells were thawed and cultured for three passages before starting the experiment. For best conditions and to ensure that cells were in an exponential growth phase when further used for animal experiments, cells were last passaged 48 h before the inoculation. On the inoculation day, cells were harvested, counted and once washed with phosphate-buffered saline (PBS; Gibco Invitrogen, Karlsruhe, Germany) to remove the remaining FBS.

### Animals

In this experimental setup, 40 eight-week-old, male, immunodeficient NOD-SCID mice (NOD.CB-17-Prkdc scid/Rj; Janvier Lab, Le Genest-Saint-Isle, France) were used. In groups of five mice were housed in a humidity- and temperature controlled 12 h dark/light environment in single ventilated cages under specific pathogen-free conditions (animal facility of the Institute for Clinical and Experimental Surgery, Saarland University, Germany). Animals had access to tap water and standard pellet food *ad libitum*. All animal experiments were approved by the local governmental animal care committee (#37-2020) and were performed in accordance with the German legislation in protection of animals, the EU Directive 2010/63/EU and the National Institutes of Health Guidelines for the Care and Use of Laboratory Animals (NIH Publication #85-23 Rev. 1985).

### Lymphogenic metastasis model

**FIGURE 2:**
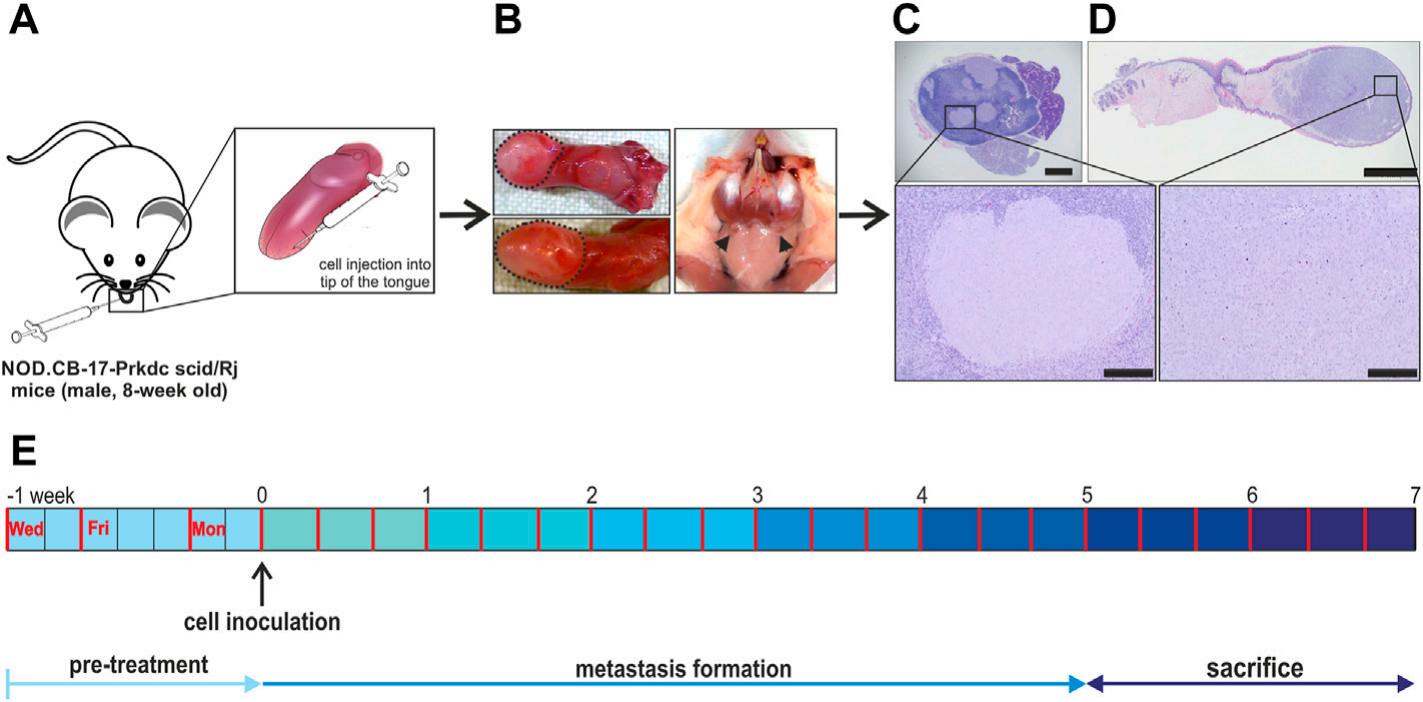
Lymphogenic metastasis model overview. **(A)** 1.0 × 10^5^ FaDu wildtype cells were injected into the right side of the tip of the tongue of 8-week-old, male, immunodeficient mice. **(B)** The sacrifice was done after 5–7 weeks of metastasis formation. Here, the tongue as well as the cervical lymph nodes were collected for further histological processing. The tongue tumor is encircled by black dashed lines and cervical lymph nodes are indicated by black arrow heads. **(C,D)** For identification and quantification of the primary tumor and metastasis the dissected tongues and related cervical lymph nodes were HE stained and further analyzed. **(E)** Schematic overview of the full experimental plan containing drug delivery 3-times per week and the sacrifice of animals after 5–7 weeks. In the beginning animals were classed into four treatment groups: TFP & TG, TFP, TG and vehicle. Scale bar: 1 mm **(C)**, 2 mm **(D)**, zoom out 200 µm.

Pharmacological treatment of all animals started 1 week before tumor cell inoculation. After 3 premature treatment days, 1.0 × 10^5^ tumor cells were inoculated into the right side of the tip of the mouse tongue at day 0 (Figures 2A,E). Therefore, mice were set under general anaesthesia (100 mg/kg ketamin, 12 mg/kg xylazin) and the mouth was opened using a self-made gag-bit. During the whole process the mice were lying on a heating plate to avoid hypothermia. The mouth was opened gently by pulling at the upper and lower incisor teeth. Using a swab and an anatomic forceps the tongue was carefully pulled out of the mouth. Tumor cells were resuspended prior loading a Hamilton syringe (Hamilton® syringe, 701N, volume 10 μL, ga26s, 51 mm; #28615-U) with 1.0 × 10^5^ cells in 5 µl PBS. The syringe was injected coming from posterior at the right side of the tongue until anterior. The syringe was guided as close as possible under the surface to the tip of the tongue where the cell suspension was injected under visual control of depot formation within the tongue tip. While pulling out of the syringe the injection channel was clamped for a few seconds to inhibit a reflux of injected cell suspension as well as to ensure a spheroid tumor growth. After cell injection mice received an analgesia depot (5 mg/kg carprofen) subcutaneously and were allowed to wake up.

### Treatment and dissection of mice

Animals were randomly divided into four groups and were treated over 5–7 weeks. Drug delivery was designed by intraperitoneal (i.p.) injections three times a week in 48 and 72 h intervals either vehicle (*n* = 9), 0.4 mg/kg TG (*n* = 10), 15 mg/kg TFP (*n* = 10) or the combination of both substances (*n* = 11) (Figure 2E). Animals that received the combination of both substances had a break of 2 h in-between drug injections to ensure valid absorption and to allow animals to recover from potential side effects from the first drug delivery. Due to drug-specific physiological effects, substance delivery was started with TFP first, followed by TG as the second substance. Mice were monitored at least three times a week, scoring general state of health, behaviour in the group, food and water intake, and body weight. At the end of the specified treatment period mice were sacrificed using an overdose of anaesthetics. Tongues containing the primary tumors and the cervical lymph nodes were carefully excised under video documentation and processed for further histological analysis (Figures 2B–D).

### Histological analysis

The cervical lymph nodes and the tongues of the mice were formalin-fixed and embedded in paraffin (Tissue-Tek^®^VIP™ 5 Jr, Sakura Finetek Japan Co., Ltd., Tokio, Japan) after 5 till 7 weeks of therapeutic treatment procedure (Figures 2B–D). Using a Leica RM 2125 RT rotary microtome (Leica Biosystems Nussloch GmbH, Heidelberg, Germany) 3 µm thick sections were cut for haematoxylin-eosin staining (HE). Tongues were cut until the mid of the tumor was reached to have the full tumor area in diameter. Lymph nodes were fully cut for evaluation of metastasis burden (Supplementary Figure S1). Every 20 µm a HE stained tissue slide was made and evaluated. Stained sections were analyzed in a double-blinded setup for primary tumor burden and lymph node metastasis by using an Olympus BX61 microscope (Olympus, Hamburg, Germany) by two investigators. Images were taken using the aforementioned BX61 microscope and a Leica MZ10 F stereomicroscope in combination with a transmitted light plate (Leica Microsystems, Wetzlar, Germany).

### Generation of SEC62-knockout cell line

For generation of the *SEC62* knockout cell line the previously described plasmid by Fumagalli et al. (2016) was used. Therefore, 2.5 × 10^6^ FaDu wild type cells were seeded into 6 cm plates 24 h before transfection. The used plasmid was based on the lentiCRISPRv2-puro system (#52961; Addgene, Watertown, MA, United States) and included already the human *SEC62* target sequence for the guide RNA (gRNA) (5‘-CTG TGG TTG ACT ACT GCA AC-3‘). The plasmid was transfected utilizing FuGene^®^ HD (Promega Corporation, Madison, WI, United States) according to the manufacturer´s instructions to generate *SEC62*-knockout FaDu cells. After another 24 h, cells were set under selection pressure using puromycin (1.5 μg/ml). Transfected cells were further cultured for 5 days prior to separation in low cell numbers of about 1000–2000 cells before single cell isolation into a 14.5 cm culture dish for another 12 weeks until potential single cell clones started to form new colonies. Those colonies were harvested and seeded into a 96-well plate for an additional single cell clone selection step (0.5 cells/well). Cells were cultured for further 8 weeks until new monoclonal colonies were formed. *SEC62*-knockout was verified by western blotting and next generation sequencing (NGS).

### Western blot

2 × 10^6^ cells were lysed in RIPA buffer (#39244.02; SERVA Electrophoresis GmbH, Heidelberg, Germany) containing protease inhibitors (#39102.01; 1:100; SERVA Electrophoresis GmbH, Heidelberg, Germany) according to manufacturer´s instructions. Subsequently, proteins were resolved by SDS-PAGE and blotted per semi-dry transfer onto a polyvinylidene fluoride (PVDF) membrane (#IPFL00005, Merck Millipore Ltd., Carrigtwohill, Co. Cork, Ireland). For detection of the total protein amount a ponceau staining was done. Target proteins were identified by antibody detection, using an affinity-purified polyclonal rabbit anti-peptide antibody for SEC62 and BiP. Here, the used SEC62 antibody was directed against the C-terminus of human SEC62 as well as the used BiP antibody was directed against the amino-terminal peptide of human BiP (1:1000 each; self-made and kindly provided by Prof. Martin Jung, Department of Medical Biochemistry and Molecular Biology, Saarland University, Homburg/Saar, Germany). As a loading control, β-actin was used (1:5000; #A5441-.2ML; Sigma-Aldrich, St. Louis, MO, United States). ECL Plex goat anti-rabbit Cy5 and anti-mouse Cy3 conjugates (1:2500; 1:2500; GE Healthcare, Munich, Germany) were used as secondary antibodies. Blots were imaged using an Intas ECL Chemocam Imager in combination with the ChemoStar image acquisition software (Intas Science Imaging Instruments GmbH, Göttingen, Germany). The SEC62 and β-actin levels were quantified and normalized using the ImageJ software (Schindelin et al., 2012).

### Targeted NGS and data processing

Genomic DNA (gDNA) was extracted from cell pellets of FaDu wt, clone 1, and clone 2 using the NucleoSpin Tissue kit (#740952.50, Macherey-Nagel GmbH, Düren, Germany). 50 µg of isolated gDNA were amplified and tagged with a unique NGS barcode (F-primer: 5´-TCT​TTC​CCT​ACA​CGA​CGC​TCT​TCC​GAT​CTG​TTG​AGG​TTT​TGG​GGA​ACT​AC-3´; R-primer: 5´-GTG​ACT​GGA​GTT​CAG​ACG​TGT​GCT​CTT​CCG​ATC​TAG​AGG​TAC​CAA​AGA​AAT​CAG​G-3´; barcode fraction is underlined) using the HotStar Taq-polymerase (#203203, Qiagen, Hilden, Germany) according to the manufacturer´s instructions. The PCR started with a denaturing step of 15 min at 95°C, followed by 35 cycles of 95°C for 1 min, the primer annealing at 60°C for 1 min 30 s and the elongation at 72°C for 45 s. Subsequently, 10 min at 72°C finished the PCR. Afterwards, all samples were purified by AMPure XP beads (#A63881, Beckman Coulter, Krefeld, Germany) and quantified by Qubit (Thermo Scientific, Waltham, MA, United States) following the manufacturer´s instructions. All generated amplicons were labelled with the same unique barcode because the samples were split into different pools before loading on an Illumina MiSeq sequencing machine (Illumina, San Diego, CA, United States). Samples were sequenced for 2 × 250 bp paired-end to nearly 200,000-fold coverage in cooperation with Dr. Tierling (Department of Genetics and Epigenetics, Prof. Walter, Saarland University, Saarbrücken, Germany). The generated data was further processed and analyzed with CRISPResso2 software (Clement et al., 2019).

### Proliferation assay

The analysis of the proliferation potential of the investigated cell populations was carried out in real-time using the xCELLigence RTCA SP system (OMNI Life Science GmbH & Co KG, Bremen, Germany) according to the manufacturer’s instructions. The working principle of the applied method lies in an electrical impedance measurement via gold electrodes at the bottom of the assay-specific e-plates. An increase in electrical impedance is generated through attachment of the seeded cells as well as proliferation of cells. Therefore, the generated signal is transferred into a unitless readout called cell index. The electrical impedance in this system indicated by the cell index is proportional to the area of the well surface covered by the cells. In brief, 0.75 × 10^5^ cells were seeded into a 96-well E-plate (OMNI Life Science GmbH & Co KG, Bremen, Germany) and were allowed to sediment for 30 min before the real-time assay was started. To determine the proliferation potential, the respective cell index was measured automatically every 15 min for at least 120 h using the associated RTCA software. In case of medication, the measurements were interrupted after 5 h of cell sedimentation for drug delivery. During the entire analysis, cells were cultivated under normal cell culture conditions. To determine the exponential growth phase the slope of the respective proliferation curve during this period was calculated using the RTCA software 2.0 (OMNI Life Science GmbH & Co KG, Bremen, Germany).

### Migration assay

Analyses of the migration potential of the investigated cell populations were carried out using the FluoroBlok migration inserts (Corning, Corning, NY, United States) and related culture plates. Therefore, the respective cell populations were seeded into migration inserts in a concentration of 0.5 × 10^5^ cells per insert. The bottom of the inserts is formed by a fluorescence-blocking membrane with 8 µm pores allowing cells to migrate through these pores. The working principle of the applied assay is based on the Boyden Chamber assay using a FCS gradient between the migration insert and the chambers of the culture plate, with FCS serving as a chemoattractant (Boyden, 1962). For the investigated FaDu cells - wildtype and generated knockout cell lines - the migration medium consisted of unsupplemented culture medium with previously added 0.1% (v/v) FCS before analysis. The migration inserts were then placed in the culture plate containing regular culture medium with an FCS content of 10%. After 72 h, the assay was stopped by a methanol-based cell fixation followed by a subsequent DAPI staining (1:1000, #MBD0015-5ML, Sigma-Aldrich Chemie GmbH, Taufkirchen, Germany) of the migrated cells. For analysis, three representing images of each insert were taken using the Nikon Eclipse TE 2000S fluorescence microscope (Nikon, Minato, Japan) and semi-automatic quantitative analysis was done using the NIS-Elements AR software (version 3.22.14; Nikon, Minato, Japan).

### Live cell calcium imaging

FaDu wt, clone1 and clone2 cell colonies were plated on poly-L-lysin coated coverslips (FaDu WT: 0.3 × 10^6^ cells/coverslip, clone1: 0.3 × 10^6^ cells/coverslip, clone2: 0.35 × 10^6^ cells/coverslip) and were cultured for 24 h in a humidified environment at 37 °C and 5% CO_2_ [Ca^2+^]_cyt_ was imaged using FURA-2 AM as previously described (Lang et al., 2011). The cells were loaded with 4 μM FURA-2 AM at room temperature for 20 min in 1 mM Ca^2+^ containing solution (140 mM NaCl, 4 mM KCl, 1 mM MgCl_2_, 1 mM CaCl_2_, 10 mM Glucose, 10 mM HEPES, pH 7.4). The loading solution was replaced by Ca^2+^-free solution (140 mM NaCl, 4 mM KCl, 1 mM MgCl_2_, 0.5 mM EGTA, 10 mM Glucose, 10 mM HEPES, pH 7.4) before starting Ca^2+^ imaging. Ca^2+^ imaging experiments were performed with an inverted microscope (Axiovert Oberver D1, Carl Zeiss, Jena, Germany) combined with an illumination system (Lambda DG-4, Sutter Instrument, Novato, CA, United States), dual CCD camera (ORCA-D2, Hamamatsu Corporation, Bridgewater Township, NJ, United States) and data acquisition VisiView software (Visitron Systems GmbH, Puchheim, Germany). FURA-2 intensities were obtained by alternated excitation at 340 nm (340/26 BrightLine HC, AHF analysentechnik AG, Tübingen, Germany) and 387 nm (387/11 BrightLine HC, AHF analysentechnik AG, Tübingen, Germany) and the emission was detected at 510 nm (ET510/80 m Chroma, AHF analysentechnik AG, Tübingen, Germany). Image pairs of FURA-2 were recorded every 3 s with an exposure time of 10 ms and 2 × 2 binning. FURA-2 signals were recorded as single cell ratios (R) of the background subtracted fluorescence intensities at 340 and 387 nm, respectively. The maximum fluorescence ratio (R_max_) was measured by adding 25 mM external Ca^2+^ in the presence of 10 μM ionomycin (IONO). 20 µM IONO in the presence of 15 mM EGTA was used to measure the minimal fluorescence ratio (R_min_). R values were converted in [Ca^2+^]_cyt_ using the equation

$${\left[Ca^{2+}\right]}_{cyt}=\beta K_{ad}\cdot \left(R-R_{min}\right)/\left(R_{max}-R\right)$$

where *βK*_*ad*_ represents the system specific apparent Ca^2+^ dissociation constant for FURA-2 as previously described (Pick et al., 2021).

Stock solution of TFP, TG and IONO were prepared by dissolving the substances in DMSO. 5 min before starting the experiments, stock solutions were diluted to a 2 times (2x) lower concentration and applied to the bath solution in a 1:1 dilution to avoid problems arising from slow mixing. The final DMSO concentration in the recording chamber was 0.1% (v/v). This concentration of DMSO was used for control experiments. We considered statistical non-significant differences in changes in the [Ca^2+^]_cyt_ that were below 25 nM, based on the analysis of the basal noise in the FURA-2 signals. Data were analyzed using commercially available softwares Excel 2010, Origin 8.6, and Sigma Plot 10.0.

### Statistics

All statistical analyses within this work were carried out using the software OriginPro (version 2021; OriginLab Corporation, Northampton, MA, United States). After data sets were tested for normal distribution with a Shapiro-Wilk test and for equal variances, differences were tested using an unpaired *t*-Test. If variances were not equal a Welch correction was done. For analyzing the *in vivo* experiments a Fisher´s exact test was used. In case of live cell Ca^2+^ imaging experiments a non-parametric two-sample Kolmogorov-Smirnov test was used. A statistical significance was achieved when the *p*-value was less than 0.05.

## Results

### TFP and TG tolerance in NOD-SCID mice

In our *in vivo* study 8-week-old, male NOD-SCID mice were inoculated with FaDu wild type cells into the right side of the tip of their tongue. Mice were treated either with TFP and TG in combination (*n* = 11), with TFP (*n* = 10) or TG (*n* = 10) as a single drug or served as a vehicle group (*n* = 9) only treated with the appropriate solvent. Within the following 5–7 weeks, the injected tumor cells generated a primary tumor in the tip of the tongue and were able to migrate into the cervical lymph nodes to generate lymphatic metastases (Figures 2A–D). Notably, two animals generated additionally tumors in the ground of the tongue despite the tumor in the tip of the tongue. The maximal test duration of 7 weeks or a loss of weight were main criteria to sacrifice the animals. In general, the drug administration caused no severe side effects as mice displayed normal fur care and movement behaviour in all groups at least 5 h after treatment. Animals showed a normal food and water intake as well as a normal respiration rate (Supplementary Figure S2). It must be noted that animals receiving TFP treatment were exhausted about 3–5 min after drug administration but were able to recover within the following 2 h. To avoid a hypothermia, the cages of treated mice were positioned in front of a heating lamp. In case of the combinatory treatment group mice were allowed to recover from the first drug administration (TFP) for at least 2 h before TG was administered as mice slowly started again to walk through the cage after that time. After TG administration there was no severe impairment of mice visible. Mice that received TG treatment as a single treatment were minimal exhausted for about 20 min and started to behave in a normal way after this time. A worsening of the symptoms due to a possible accumulation of the substances by repeated administration could not be determined. Taken together, the administration of the two substances–TFP and TG -, in combination and alone, was tolerated by the used mouse strain and no acute severe side effects or additionally chronic side effects because of drug accumulation were registered.

### TFP suppresses metastasis rate and TG reduces metastases size of HNSCC *in vivo*

**FIGURE 3:**
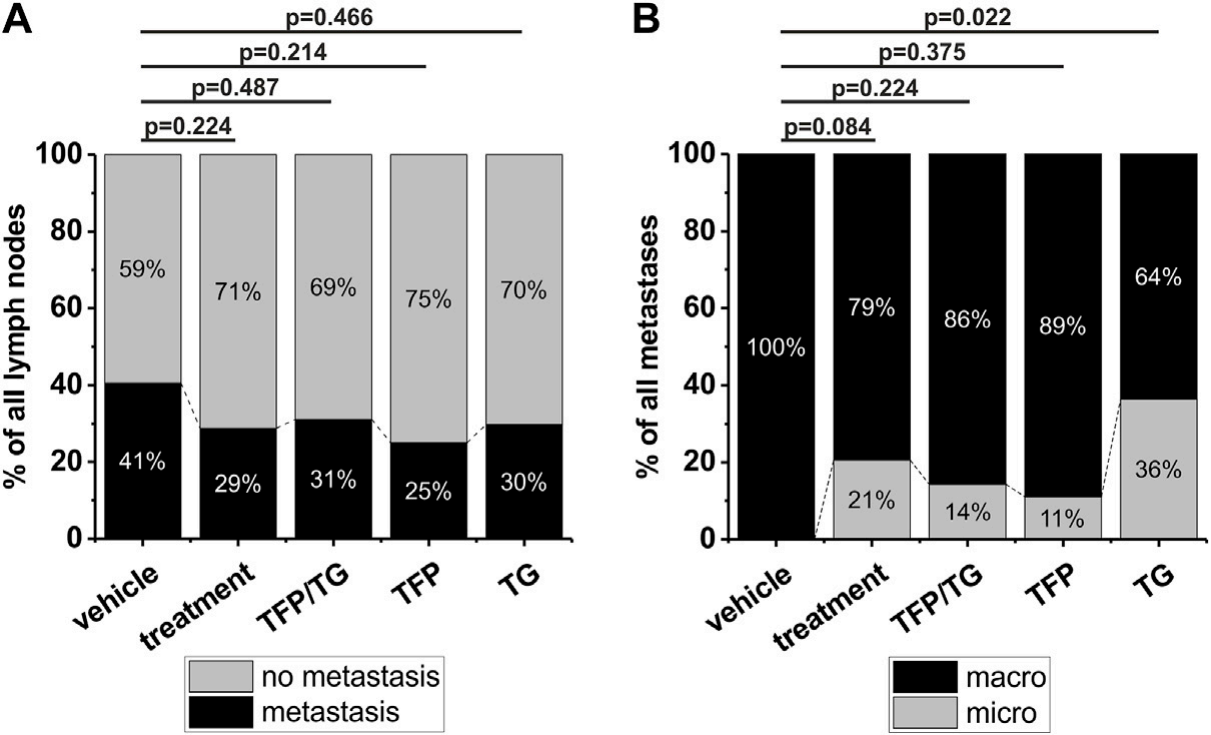
Impact of different drug treatments on metastasis growth and metastasis size in 40 NOD-SCID mice. Groups compared to each other are vehicle (*n* = 9), treatment (*n* = 31) that includes all treatments in general, combinatory treatment TFP/TG (15 mg/kg TFP, 0.4 mg/kg TG) (n = 11), 15 mg/kg TFP (*n* = 10) and 0.4 mg/kg TG (n = 10). After 5–7 weeks of drug delivery three times per week animals were sacrificed, tongues and cervical lymph nodes were dissected and further processed for histological analysis including HE staining. **(A)** Comparison of the number of detectable metastases of all dissected lymph nodes in vehicle animals versus the above-mentioned groups. Animals which received TFP and TG in a combination therapy, got drugs with 1 h in-between drug delivery. **(B)** Comparison of the detectable metastasis size in vehicle animals versus the aforementioned groups. Significance was tested using a Fisher´s exact test.

As mentioned above the injected tumor cells were able to migrate into the cervical lymph nodes to generate lymphatic metastases (Figures 2A–D). Based on the used number of animals here, a slight decrease in the number of detected metastases was observed for all treated animals in general compared to the vehicle group (reduction of 12%, *p* = 0.224, Supplementary Table S1). Animals which were treated with the combinatory treatment of TFP and TG showed a reduction in the number of detected metastases of 10% (*p* = 0.487; Figure 3A). In this group in total 45 lymph nodes were dissected, and 14 lymph nodes were diagnosed with metastases. In case of the solo TFP treatment group in sum 36 lymph nodes were dissected from which 9 lymph nodes showed metastases. In this case the animals showed a decrease of metastasis in general of 16% (*p* = 0.214; Figure 3A). Animals which were treated with TG alone showed a reduction in the number of detected metastases of 11% (*p* = 0.466; Figure 3A). Here, a total of 37 lymph nodes were dissected, of which 11 lymph nodes showed metastases.

Next, the size of metastasis was examined and compared between the different treatment groups. Based on clinical parameters metastases consisting of more than 20 tumor cells were termed as macro metastases and all metastases consisting of less than 20 tumor cells were termed as micro metastases. All detected metastases of vehicle animals were categorized as macro metastases (*n* = 15). In case of all three treatment groups in general 21% of all detected metastases were micro metastases (*p* = 0.084; when compared to the vehicle group; Figure 3B). For the combinatory treatment of TFP and TG a total of 2 out of 14 metastases were categorized as micro metastases, that results in 14% micro metastases 0.224; *p* = Figure 3B). For TFP solo treatment 1 out of 8 metastases was categorized as a micro metastasis, which results in 11% micro metastases (*p* = 0.375; Figure 3B) and for TG, 4 of 7 metastases were categorized as micro metastases (Figure 3B). The treatment administering TG as a solo drug the micro metastases identification rate increased significantly to 36% (*p* = 0.022). Taken together, we found a reduction in number of lymph node metastases by TFP treatment as well as a reduction in metastases size by TG treatment in our newly established orthotopic xenograft *in vivo* mouse model.

### CRISPR-Cas9 mediated SEC62 full knockout

**FIGURE 4:**
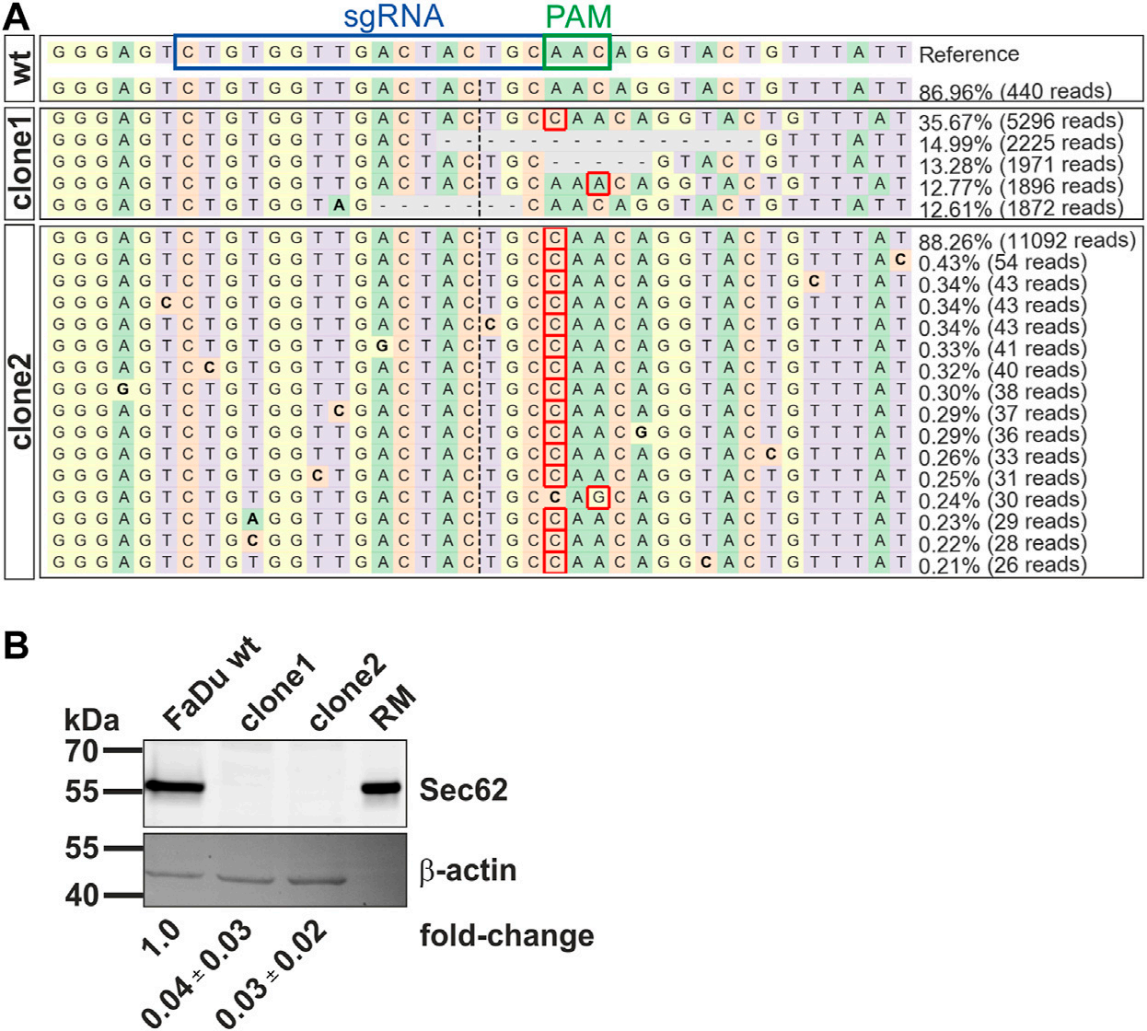
Characterization of newly generated *SEC62*-knockout FaDu cell clones by CRISPR-Cas9 event. **(A)** Visualization of identified alleles by NGS around the Cas9 cleavage site. Each sequenced nucleotide was marked by a unique colour (A: green, C: red, G: yellow, T: purple). Insertions were marked with a red rectangle; substitutions were highlighted in bold, and deletions were marked with horizontal dashed lines. First the reference genome including the used sgRNA (blue box) and the related PAM-sequence (green box) was shown. Then the results generated by CRISPResso2 analysis of the wt sequencing and the sequencing of both cell clones were listed. **(B)** Cellular protein levels of SEC62 were quantified in FaDu wt cells and *SEC62*-knockout cell clones. Under the respective bands the SEC62 fold-change is indicated as a mean value (*n* = 3) with the respective standard deviation. RM, rough microsomes.

For further investigation of the role of SEC62 in HNSCC, especially in the proliferation and migration behaviour of HNSCC cancer cells, an immortalized cell line of a hypopharyngeal squamous cell carcinoma (FaDu) was used. Here, a full *SEC62* gene knockout was generated by the CRISPR-Cas9 technology. For CRISPR event validation FaDu wt cells as well as two selected cell clones were sequenced by NGS. Previously, Bochen et al. (2017) showed an amplification of *SEC62* on chromosomal as well as on protein level (Bochen et al., 2017). Based on these facts Cas9 has to be active for several times. After NGS, data sets were analyzed using the CRSIPResso2 platform (Clement et al., 2019). Here, after a pre-processing step, the obtained fastq-files were aligned against the human genome and various alleles in each clone were identified. In case of FaDu wt, 87% (440 reads) of the sequencing reads matched to the reference genome (Figure 4A). For clone 1 there were 5 different alleles identified after the Cas9 activity (Figure 4A). These 5 newly generated alleles count for 88.5% of all analyzed reads (13,260 reads). The most abundant allele in clone 1 had an insertion of a cytosine in front of the PAM sequence (36%). The other alleles mostly consisted of a deletion of 5 up to 15 nucleotides. In clone2 differ 92.1% (11,644 reads) of all reads from the reference genome, mainly containing a cytosine-insertion in front of the PAM-sequence. After the CRISPR-Cas9 event validation on nucleotide base the Cas9-caused alterations were also analyzed on protein level. Setting the SEC62 signal for FaDu wt as a reference, both clones were analyzed for their remaining SEC62 protein content via western blot using β-actin as a loading control. In both cases, remaining protein content was nearly undetectable (clone1: 0.04 ± 0.03; clone2: 0.03 ± 0.02; Figure 4B). In summary, we were able to generate two new cell lines using the CRISPR-Cas9 technology which display a *SEC62* knockout and could be validated by NGS and western blot.

### Reduced proliferation and migration in SEC62-knockout cells

**FIGURE 5:**
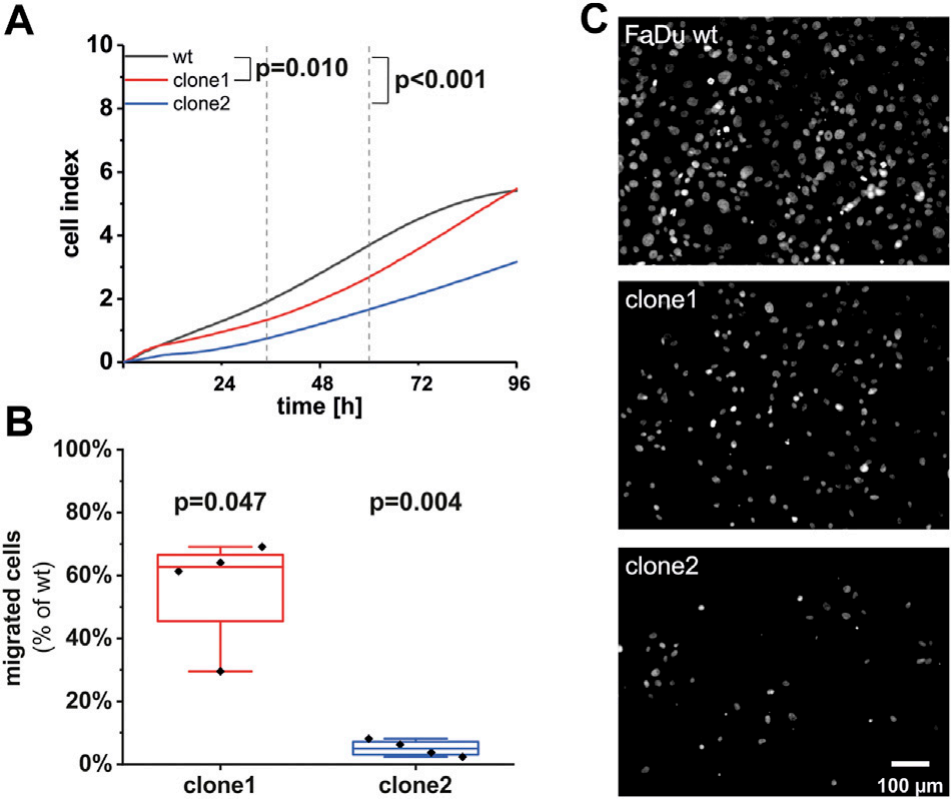
Effect of the *SEC62*-knockout on the proliferative and migratory potential of newly generated FaDu cell clones. **(A)** As an indicator for cell proliferation in real-time the unitless cell index of FaDu wt (black line), clone 1 (red line) and clone 2 (blue line) was measured for 96 h. Shown is the mean of measured cell indices (*n* = 12). Each experiment was performed in quadruplicates for each cell population. The slope of each cell line was calculated within a time range of 35 h up to 60 h after cell seeding. The proliferation rates of clone 1 (*p* = 0.0095) as well as clone 2 (*p* = 3.34e-4) were significantly reduced compared to the wt cells. **(B)** Cells were allowed to migrate through 8 µm pores of the insert system for 72 h. Each experiment was performed in technical duplicates, biological replicates were done 4 times. For quantification 3 different areas per insert were analyzed. Shown is the percentage of migrated cells of both clones in comparison to wt cells. The line within the box indicates the median. **(C)** Representative images of migrated cells of different cell populations. Scale bar: 100 μm wt, wild type.

To determine whether different expression levels of *SEC62* due to the *SEC62* knockout affect HNSCC cell biology, we performed functional analyses using FaDu wt and the two aforementioned CRISPR-Cas9 cell clones. First, we analyzed their proliferative potential using the xCELLigence system (OMNI Life Science GmbH & Co KG, Bremen, Germany). For wt and clone1 we observed a simultaneous proliferation in the first 10 h of the test which started to differentiate after this time range. In contrast, clone2 differed in its proliferation pattern compared to wt during the whole observation period. After calculation of the slope of the determined cell indices in a time range from 35 to 60 h representing the exponential growth phase for all three cell lines, we found a significantly reduced proliferation rate of both clones compared to wt cells (clone1 *p* = 0.0095; clone2 *p* = 3.34e^−4^). Afterwards we investigated their migratory potential using the FluoroBlok system (Corning Inc, New York, United States) and observed a markedly reduced migratory potential in both examined clones compared to wt (Figure 5B). Representative images of migrated cells after fixation and staining with DAPI are shown in Figure 5C. Altogether, we found that *SEC62* knockout resulted in a decreased proliferation and migratory potential (Figure 5).

### Effect of *SEC62*-knockout on cellular Ca^2+^ homeostasis

**FIGURE 6:**
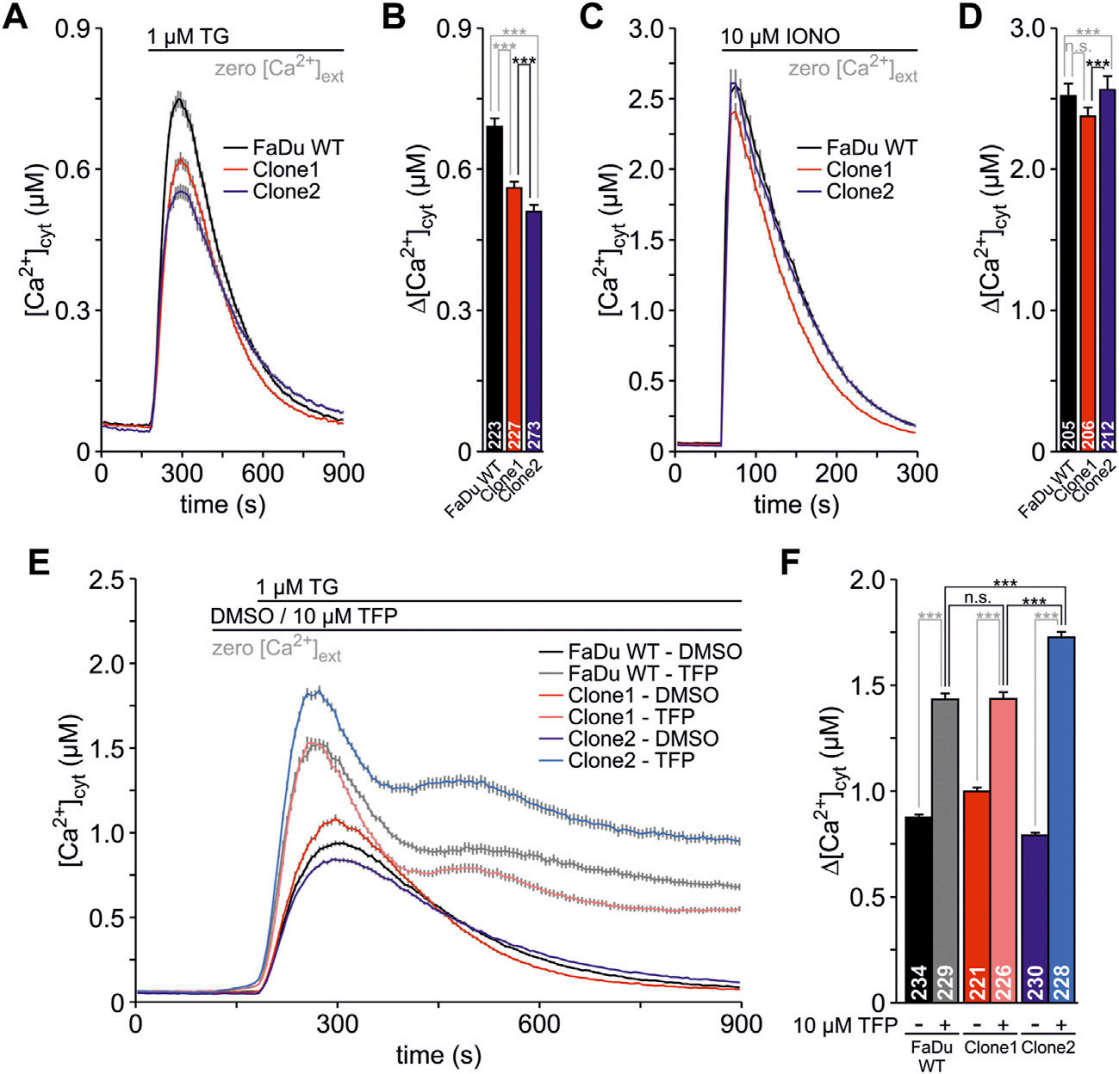
Live cell Ca^2+^ imaging of FaDu wt and *SEC62*-knockout clones. The responses of the cells to TG and IONO were visualized by detecting the cytosolic Ca^2+^ concentration ([Ca^2+^]_cyt_) with FURA-2. The cells were seeded onto poly-L-lysin coated coverslips 24 h prior to the live cell Ca^2+^ imaging experiments. **(A)** The Ca^2+^ leak from the ER was unmasked using 1 μM TG in absence of extracellular Ca^2+^ (zero [Ca^2+^]_cyt_) **(B)** The TG responses were analysed by the increase from the basal to the maximal [Ca^2+^]_cyt_ and are illustrated as Δ[Ca^2+^]_cyt_ **(C)** The Ca^2+^ mobilisation from internal stores was induced by the application of 10 µM IONO in the presence of zero [Ca^2+^]_cyt_ to evaluate the total Ca^2+^ content of the cells **(D)** The IONO responses were expressed as Δ[Ca^2+^]_cyt_ **(E)** During the measurements, cells were treated with 0.1% DMSO (as a control) or 10 µM trifluoperazine (TFP) for 1 minute. Subsequently, 1 μM TG was used to unmask the Ca^2+^ leak from the ER **(F)** The responses of the different treatments are shown by Δ[Ca^2+^]_cyt_. Data is shown as mean ± SEM. The total number of analysed cells from three independent experiments is indicated within graph bars for each cell line. n.s., not significant; ***, *p* < 0.001.

In addition to the functional analysis of the newly generated *SEC62*-knockout clones in terms of their migratory as well as their proliferative behaviour we also investigated their cellular Ca^2+^ homeostasis using FURA-2 as previously described (Lang et al., 2011; Pick et al., 2021). The measurements were carried out in absence of extracellular Ca^2+^ (zero [Ca^2+^]_cyt_) in order to detect only the Ca^2+^ leak from the ER. In this experimental setup, all tested cell lines showed no differences in the basal Ca^2+^ level (Figure 6A). To unmask the Ca^2+^ leak from the ER, 1 μM TG was used which showed a robust increase of the [Ca^2+^]_cyt_ in all three cell lines. The quantified TG responses represent the increase from the basal to the maximum [Ca^2+^]_cyt_ (Δ[Ca^2+^]_cyt_) where the FaDu wt cells showed the strongest Ca^2+^ leak (Δ[Ca^2+^]_cyt_: 0.69 ± 0.018 µM, Figure 6B). Comparable to the other functional analyses clone2 (Δ[Ca^2+^]_cyt_ = 0.51 ± 0.014 µM) showed a higher difference in Δ[Ca^2+^]_cyt_ compared to clone1 (Δ[Ca^2+^]_cyt_ = 0.56 ± 0.013 µM). In a next step we investigated the total amount of cellular Ca^2+^ within all 3 cell types by the application of 10 µM IONO (Figure 6C). We saw statistically significant differences between the total Ca^2+^ amount when comparing all tested cell lines (Figure 6D), however, those differences seem to be too small (FaDu wt: Δ[Ca^2+^]_cyt_ = 2.52 ± 0.088 µM, clone1: Δ[Ca^2+^]_cyt_ = 2.38 ± 0.061 µM, clone2: Δ[Ca^2+^]_cyt_ = 2.56 ± 0.096 µM) to have an effect on the Ca^2+^ leak so that we do not expect any biological relevance of this observation. Since it is known that TFP increases the Ca^2+^ leak through Sec61 complexes in various cell types (Erdmann et al., 2011; Linxweiler et al., 2013; Bhadra et al., 2021; Pick et al., 2021), we also tested the effect of TFP in the FaDu wt and *SEC62*-knockout clones (Figure 6E). After exposure of 10 µM TFP or 0.1% DMSO for 1 minute, 1 μM TG was added to the cells. The pre-treatment with TFP led to a significant increase of [Ca^2+^]_cyt_ in all three cell lines. This increase was significantly augmented after the additional administration of TG (Figure 6F). Here, clone2 (Δ[Ca^2+^]_cyt_ = 1.73 ± 0.026 µM) showed the highest and most rapid increase of cytosolic Ca^2+^ whereas clone1 (Δ[Ca^2+^]_cyt_ = 1.44 ± 0.032 µM) and wt (Δ[Ca^2+^]_cyt_ = 1.43 ± 0.029 µM) were not statistically different. In general, we observed a rather long time period for Ca^2+^ clearance after TFP treatment (Figure 6E), which seems to be characteristic for the investigated FaDu cell line and which is different to other human cell lines e.g. HeLa, HCT-116, RPMI 8226 and NALM-6 (Erdmann et al., 2011; Linxweiler et al., 2013; Bhadra et al., 2021; Pick et al., 2021).

In summary, Ca^2+^ imaging experiments showed that *SEC62*-knockout clones have a rather similar general cellular Ca^2+^ content compared to FaDu wt cells. However, the generated mutation by Cas9 cutting in clone2 has a greater impact in sensitizing the cells towards TG and TFP compared to clone1 and wt cells.

### *SEC62*-knockouts affects HNSCC cell resistance to TG and TFP

**FIGURE 7:**
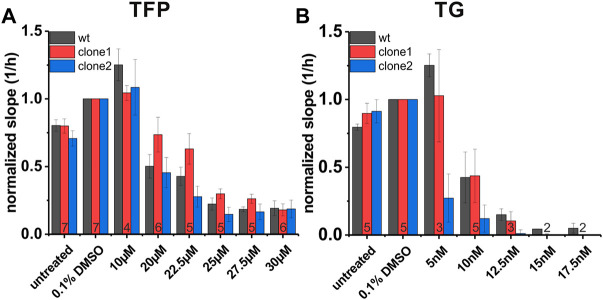
Proliferation behaviour of FaDu wt cells and *SEC62*-knockout cell lines under TFP and TG treatment. For data analysis the slope of each cell line was calculated within a time range of 35 h up to 60 h after cell seeding. Treatment was started 5 h after cell seeding. All measurements were made in technical quadruplicates, biological replicates were indicated at the bottom of the red column. Shown is the mean of the slope (1/h) normalized to solvent control (0.1% DMSO). The error bars indicate the standard error of the mean **(A)** Proliferative behaviour of three different cell lines under TFP treatment **(B)** Proliferative behaviour of the same cell lines under TG treatment. FaDu wt cells (black bars), clone 1 (red bars) and clone 2 (blue bars). wt, wild type.

Next, we analyzed the impact of *SEC62* knockout on cell proliferation under specific drug treatments which are known to cause ER stress. For this purpose, we used both substances TFP and TG, that we also tested in our lymphogenic *in vivo* model, by the same experimental setup as described for the clone characterization using the xCELLigence system. One difference in the experimental setup was that the drug administration started 5 h after cell seeding, to allow cells to sediment before the drug was added. The slope was calculated for all three cell lines in a time range of 35–60 h after cell seeding within the exponential growth phase of the wt cells. All obtained results were compared to the control group (0.1% DMSO). In both treatment groups differences between both tested clones in their sensitivity to TFP and TG were observed (Figure 7). In general, clone1 seemed to be more resistant regarding drug administration compared to clone2. For TFP treatment clone1 was impaired in its proliferative behaviour by 50% at a concentration of 25 µM (cell index 0.298). In case of the wt cells and clone2 this inhibition of cell proliferation was already visible at 20 µM (wt = 0.502, clone 2 = 0.455; Figure 7A). Concentrations of TFP higher than 25 µM seemed to impair cells in their ability to proliferate even further. For TG we could recognize a straight decrease in proliferation for clone2 at a TG concentration of 5 nM (0.27; Figure 7B). Wt cells and clone1 proliferated at this concentration with a cell index of 1.25 (wt) and 1.03 (clone1). Doubling the TG concentration to 10 nM wt cells and cells of clone1 also rapidly decreased in their ability to proliferate (wt = 0.425, clone1 = 0.437). At a TG concentration of 12.5 nM nearly no remaining proliferation was detectable for all 3 cell lines. These experiments showed that different CRISPR-Cas9 events causing a *SEC62*-knockout led to different characteristics in proliferation behaviours after ER stress induction by TFP or TG administration.

## Discussion

In this study we established an orthotopic HNSCC murine xenograft model to investigate two potentially anti-proliferative and anti-metastatic therapeutics as a novel approach for targeted tumor therapy in head and neck cancer. In detail, the new murine model was used to investigate the effects of a calmodulin-antagonist named TFP and a SERCA pump inhibitor called TG on proliferation and migration of HNSCC cells *in vivo*. HNSCC belong to the sixth most common cancer entities worldwide and patients show a poor clinical outcome that has not been significantly changed over the past decades (Fakhry et al., 2017; Bray et al., 2018; Horton et al., 2019). Also because of a lack of disease specific therapeutic strategies in the last decades we investigated a new possible strategy to target the *SEC62* oncogene that is highly overexpressed in 86% of all HNSCC patients (Wemmert et al., 2016). We were able to establish a reproducible orthotopic and xenograft murine model for lymphogenic metastasis. This new model enabled us to test the aforementioned new potential cancer therapeutics *in vivo*.

Therefore, we inoculated immunosuppressive NOD-SCID mice HNSCC cells at the orthotopic location of the patient´s primary tumor site of the tongue. Compared to other studies using a lymphogenic metastasis model we performed all *in vivo* studies with male mice to mimic the overall clinical situation (Heath et al., 2012; Ferlay et al., 2019). Also the age of used mice differed in these studies from 4- to 12-week-old mice (Myers et al., 2002; Gleysteen et al., 2008; Sano et al., 2011; Heath et al., 2012). In our study all *in vivo* experiments were performed with mice at the age of 8 weeks to be as constant and comparable between single experimental runs as possible. Moreover, other studies showed a large variability in the number of injected tumor cells between 2-fold up to 25-fold compared to our inoculation tumor cell number of 1.0 × 10^5^ (Myers et al., 2002; Gleysteen et al., 2008; Sano et al., 2011; Heath et al., 2012). Additionally, the injection volume of the tumor cell suspension varied largely among the aforementioned studies from 25–50 μl, which is in fact 5–10-fold higher than our administered injection volume of 5 µl. We have chosen this comparable low total injection volume in order to establish a less invasive and painful tumor cell inoculation procedure with a minor impairment of inoculated mice for food intake in the following days. The incidence and number of lymph node metastases reported in animal studies using the same disease model were comparable to the results of our study (Sano et al., 2011; Masood et al., 2013).

Using this established model we tested two new potentially anti-cancer drugs with already promising results in *in vitro* assays as well as in a primary *in vivo* study (Linxweiler et al., 2012, 2013; Körbel et al., 2018). Therefore, mice injected with human HNSCC cells were treated every 48–72 h with TFP and TG in combinatory as well as in solo drug administration and the appropriate solvent reagent as negative control. Especially with regard to a potential clinical application of TFP and TG, one of our most important results was a high tolerability of both substances, in combinatory and solo administration. Neither severe acute side effects on the used mouse strain nor long-term side effects were recognized in the time range of therapy of about 5–7 weeks before the mice were sacrificed. Even mice showed an impairment in their movement 5 min after TFP administration, they were able to recover within the next 2 h. All mice were able to fully recover within 24 h before the next drug administration after 48–72 h was done. Because of the high cytotoxicity reported for TG *in vivo* (Denmeade et al., 2003; Doan et al., 2015; Jaskulska et al., 2021) a careful titration of the applied drug dose has been done before the experimental start (data not shown). Körbel et al. (2018) used a TG concentration of 1.6 mg/kg to treat athymic nude mice in their study for anti-proliferative treatment of a subcutaneously growing HNSCC flank tumor (Körbel et al., 2018). In our toxicity experiments this TG concentration caused severe side effects in the used NOD-SCID mice and ultimately resulted in death of the animals. Therefore, we decided to minimize the dose to 0.5 mg/kg. However, we significantly increased the used dose of TFP to 15 mg/kg compared to one reported by Körbel et al. (0.5 mg/kg) as various studies investigated much higher TFP doses to receive therapeutic effects that were still well tolerated by the animals (Park et al., 2016; Feng et al., 2018; Kuo et al., 2019; Qian et al., 2019; Xia et al., 2019). Both drugs were administered via intraperitoneal injection to achieve constant serum levels throughout the whole treatment phase of the experiment (Körbel et al., 2018).

Our animal experiments showed a trend for an overall suppression of lymph node metastases formation after TFP administration. This is in line with an *in vivo* study of a colorectal cancer model (CRC) reporting an efficient control of tumor growth by TFP administration (Qian et al., 2019). These authors postulated an impairment of migration and invasion by a suppression of the epithelial-mesenchymal transition (EMT). We were also able to detect a highly significant impairment in FaDu wt cell migration *in vitro* (Supplementary Figure S3). Further on, the single administration of TG showed a significant reduction of the metastasis size. This size reduction in tumor lesions by TG therapy was also observed in a flank tumor model by Körbel et al. (2018). Although these effects are comparable one has to mention that the administered TG concentration by Körbel et al. was more than 3-fold higher than the concentration administered in our study. To the best of our knowledge, a combination of both substances has not been investigated *in vivo* so far. In our present study, we found no additive effect of a combinatory treatment with TG and TFP on the number and size of the lymph node metastases, which was indicated by our previously published *in vitro* data (Linxweiler et al., 2012; Bochen et al., 2017; Supplementary Figure S3). In theory, we would have expected an additive effect of both substances on size and number of lymph node metastases due to less efficient till no recruitment of CaM under TFP administration and an irreversible inhibition of the SERCA pump by TG administration. Both mechanisms should force a higher Ca^2+^ leakage from the ER lumen to the cytosol as already seen in our live cell Ca^2+^ imaging experiments and thus generate a more efficient inhibition of HNSCC cell proliferation and metastasis as compared to a therapeutic action with the single substances. Potential explanations for this non-additive effect observed in our animal studies are a suboptimal dosing of both substances, a potential bias in histopathological analysis by missing small lymph node metastases, a potential bias in surgical excision of the very small lymph nodes by leaving single nodes in site, and compensatory cellular mechanisms like other affected Ca^2+^ leakage channels that effectively counteract a synergistic action of TG and TFP *in vivo* due to the long time of 2 h in between both drug administrations. Additionally, we have to state that the number and size of lymph node metastases showed a comparably high inter-individual variance not only in the treatment but also in the vehicle mice, so that further experiments with a higher amount of animals will be necessary to (i) confirm the observed effect of TG and TFP as mono-therapeutics on number and size of lymph node metastases and (ii) to address if there is a concentration and time dependent additive effect of both substances as combined therapy approach. Furthermore, it would be interesting to perform the animal experiments with another e.g. HPV-positive cell line to evaluate a different therapeutic effect of TG and TFP in HPV-related and HPV-independent disease. However, due to the 3R regulations we restricted our study design to one *in vivo* investigated cell line with the option of expanding the study to a second cell line when therapeutic effects are observed in this first pilot-study. Those experiments are planned for the near future.

Furthermore, we performed *in vitro* studies using the CRISPR-Cas9 technology to generate two *SEC62* knockout HNSCC cell lines. These were functionally characterized especially for the relevance of SEC62 in HNSCC and its role in cancer cell proliferation and migration. For the CRISPR-Cas9 event, we investigated the same cell line as used for the *in vivo* study which is characterized by a high overexpression of *SEC62* due to an amplification of the 3q26.2 region (Linxweiler et al., 2012; Wemmert et al., 2016; Körbel et al., 2018). In tumors overexpressing *SEC62* elevated SEC62 protein levels were associated with an increased migration and invasion potential, a higher tolerance for ER-stress and a lower sensitivity for ER-stress induced cell death (Supplementary Figure S4). From the molecular background, there are three hypotheses why tumor cells with elevated SEC62 levels show a higher resistance to ER-stress conditions. First, because of its role in protein translocation elevated SEC62 levels could help tumor cells to recover from defects in the regulation of this process i.e. the accumulation of misfolded proteins in the ER by facilitating a more efficient intracellular protein transport (Zimmermann et al., 2006). A second hypothesis is based on the role of SEC62 in recovER-phagy. Here, SEC62 functions as an autophagy receptor and under conditions of ER-stress selectively delivers ER components to the autolysosomal system and thus mediates maintenance and recovery of ER homeostasis (Fumagalli et al., 2016). This process of recovER-phagy is highly characteristic for tumor cells expressing high amounts of SEC62 and therefore distinguishes tumor cells from surrounding cells and makes tumor cells potentially accessible for SEC62 targeted treatment (Bergmann et al., 2017). A third hypothesis is based on the role of SEC62 in the regulation of cellular Ca^2+^ homeostasis. A previous study of our group could show that the migration stimulating effect as well as the better tolerance of ER-stress in *SEC62* overexpressing cancer cells can be inhibited by *SEC62* silencing, which was shown to be a Ca^2+^ dependent process (Linxweiler et al., 2013). Here, Linxweiler et al. postulated a direct regulation of Ca^2+^ efflux through the SEC61 channel by SEC62. SEC62 could be able to sense emitting Ca^2+^ by its EF hand motif and then can recruit CaM which is then closing the SEC61 channel also in a Ca^2+^ dependent way (Linxweiler et al., 2013). A mutation in the EF hand motif of SEC62 led to the same effects as a transient *SEC62* knockdown (Linxweiler et al., 2013). Therefore, the regulation of Ca^2+^ homeostasis could also be a potential point of action for a targeted therapy without directly inhibiting the SEC62 protein itself. Elevated SEC62 is able to counteract SERCA pump inhibitors like TG due to an improvement in SEC61 channel closure by efficient CaM recruitment and thus decreases cytosolic Ca^2+^ levels (Linxweiler et al., 2013). In this context, an additional inhibition of CaM is able to circumvent the rescuer role of SEC62 because its recruitment of CaM is blocked (Supplementary Figure S1). Based on this hypothesis, we established our new therapy approach to inhibit tumor cell migration by a combinatory inhibition of two important processes that essentially control cellular Ca^2+^ homeostasis at the level of the ER membrane: first, we block CaM by TFP, which leads to an elevated Ca^2+^ leakage from the ER because of an inefficient closure of the SEC61 channel. To further strengthen the effect of induced ER-stress the SERCA pump is blocked by TG. These two blockades should inhibit tumor cell migration in SEC62 overproducing tumor entities as a consequence of highly induced ER-stress by elevated cytosolic Ca^2+^ levels. *In vitro*, both substances showed a highly efficient and dose-dependent inhibition of cell proliferation and migration in various human cancer cell lines with an additive effect when administered in combination (Linxweiler et al., 2012, 2013; Supplementary Figure S3). The administered TFP and TG concentrations in solo therapy showed an even slight impairment of migration whereas the combination led to a full inhibition of the migratory potential as already suggested (Supplementary Figure S3A). The results of the western blot analysis indicate an induction of ER stress marker BiP under TG treatment as well as the combinatory treatment of both substances, that could be a first hint for higher cellular stress and therefore an impairment in migration (Supplementary Figure S3B).

However, many of the upper mentioned results were obtained using a transient siRNA based *SEC62* knockdown, which is no option for a therapeutic approach in humans and cannot be used to evaluate the role of *SEC62* expression level on tumor growth and metastasis as well as potential therapeutic interventions *in vivo*. Therefore, we generated two stable *SEC62*-knockout cell lines using the CRISPR-Cas9 technology. Both new generated cell lines showed a significant reduction in proliferation together with a significant reduction in migration consistent with the reported siRNA based *SEC62* knockdown experiments in HNSCC cell lines (Bochen et al., 2017). When comparing the biological behaviour of both cell lines with each other, we observed slightly different phenotypes in each assay, which we assume to be related to individual CRISPR-Cas9 events including also off-target effects. In the first clone its different *SEC62* alleles showed deletions of up to 15 nucleotides. Clone 2 had mainly a frameshift mutation caused by a cysteine insertion which leads to a premature stop codon at amino acid 102 in addition to an altered amino acid sequence starting at amino acid 82 near the Cas9 cutting site. Compared to the full length SEC62 protein of 399 amino acids, clone 2 shows a compliance only in the first 21% of the amino acid sequence. The residual amino acid sequence is modified by a frameshift. Therefore, we conclude that the resulting SEC62 protein is not able to perform the same functions as the wild type SEC62, which supports the results of the migration and proliferation experiments. Because we used an amplicon based NGS we were not able to see the full sequence of modified *SEC62* so that we only can speculate about the further residual protein looks like. The impairments in proliferation and migration for clone2 were much stronger compared to clone1. Here, we were not able to reliably analyze the full effect of Cas9-activity because the phenotypes of different alleles were more variable compared to clone2. Nevertheless, the deletions seem to be less effective for the destruction of SEC62 functions, because the behaviour concerning proliferation and migration of clone1 is more comparable with the wild type protein. However, western blot analysis revealed no residual protein in both clones. Both clones showed a normal viability so that apoptosis induction can be excluded as a relevant cofounder of the observed effects on migration. The growth curve measured by cell index reached a plateau phase and showed no decrease that would have been expected in case of an elevated apoptosis rate. Ca^2+^ imaging experiments revealed a similar overall cellular Ca^2+^ content of all 3 investigated cell lines. With regard to the behaviour of the 3 cell lines in response to the applied substances TFP and TG, clone2 showed a significantly increased sensitivity compared to the other two cell lines. This observation mirrors the other functional assays investigating cellular migration and proliferation where clone2 reacted stronger to TG and TFP than wt and clone1, respectively. Interestingly, the FaDu cell line seemed to be very slow in its Ca^2+^ clearance after the application of TFP in combination with TG, which has to be investigated in more detail in further experiments.

In a second step, both *SEC62* knockout clones were further analyzed and characterized concerning proliferation behaviour under ER-stress induction through TFP and TG. Especially clone2 showed the expected response of increased sensitivity for drug-induced ER-stress. Here, impairment in proliferation was induced at lower concentrations of administered drugs compared to wild type cells, in particular concerning TG administration. Regarding clone1 we generated a more resistant clone in TFP administration compared to the wild type. One potential explanation for this observation may be potential off-target effects that can circumvent the TFP medication or facilitate faster adaption mechanisms like a forced recovER-phagy or a more efficient UPR activation that can also be induced by other proteins in addition to SEC62. Qian et al. described an upregulation of LC3II-b and p62 in CRC cells after TFP treatment, which leads to autophagy induction (Qian et al., 2019). Another fact we have to consider when interpreting our results is that TFP is not only effective to CaM but also CaM has different roles within the cytosol and deals as a multifunctional intermediate calcium-binding messenger protein (Stevens, 1983). Also a compensatory effect through upregulation of plasma membrane Ca^2+^-dependent channels like NCX or PMCA could be an explanation for a less effective *SEC62*-knockout phenotype of clone1 compared to clone2 (Mohamed et al., 2010; Chou, Ju and Pan, 2015). An additional sign for a compensatory mechanism to counterbalance cellular stress induced by Ca^2+^ leakage is also visible in the Ca^2+^ imaging experiments. Here, clone1 showed no significance in the total cellular Ca^2+^ content, the same sensitivity to TFP and the same time course of TG responses. It should be mentioned that in general the FaDu cell line differs concerning the Ca^2+^ clearance significantly to other human cell lines, e.g., HeLa, HCT-116, RPMI 8226 or NALM-6 (Erdmann et al., 2011; Linxweiler et al., 2013; Bhadra et al., 2021; Pick et al., 2021).

A highly relevant advantage of both small molecules investigated in our study is the fact that TG and TFP have been used in humans either in clinical routine or clinical studies, so that detailed data on pharmacokinetics, pharmacodynamics, and adverse effects are available: Several studies addressed a potential use of TG and TG-prodrugs (e.g., G-202, 12A-DT-Asp) in cancer treatment over the past years (Denmeade et al., 2003; Christensen et al., 2009; Denmeade et al., 2012). In one first phase I trial patients with locally advanced and/or metastasized solid cancers were treated with TG-prodrug G-202 („Dose-Escalation Phase 1 Study of G-202 in patients with advanced solid tumors, Clinical Trials gov. Identifier NCT01056029). The study was closed in 2013 and showed a stabilization of clinical disease stages with an acceptable spectrum of mild to moderate adverse events (Mahalingam et al., 2016). The second small molecule investigated in our study, TFP, is established in clinical practice for treating psychiatric patients and is primarily used as part of an antipsychotic treatment of schizophrenia patients (Koch et al., 2014). A potential use of TFP in cancer treatment was investigated in first preclinical studies in recent years. Yeh et al. could show that TFP suppresses the proliferation of lung cancer stem cells and sensitized these cells to a chemotherapeutic treatment with Cisplatin (Yeh et al., 2012). Another study was able to show that TFP is able to strengthen ionizing radiation via inhibition of the repair of DNA double-strand breaks and an induction of a G2/M arrest (Gangopadhyay et al., 2007). A TFP treatment of prostate cancer and fibrosarcoma cells *in vitro* induced an inhibition of angiogenesis and a markedly reduction of tumor cell invasion, which further indicates a potential anti-metastatic effect of this substance (Pulkoski-Gross et al., 2015). This hypothesis is supported by a retrospective study on 19 patients with advanced stages of lung or colorectal cancers who were treated with calmodulin-antagonists due to a synchronous psychiatric disorder or arterial hypertension (Zacharski et al., 1990). Two of the 19 included patient were treated with TFP and showed a markedly longer overall survival than statistically expected for this stage of disease. So far, no other clinical study has investigated a potential use of TFP for cancer treatment. A combined treatment with calmodulin-antagonists and SERCA pump inhibitors has not been investigated in clinical trials so far.

Within this study we could show a tendency in the therapeutic effects of our new described targeted therapy approach using two therapeutics which leads to immense ER-stress and therefore causes cell death to reduce metastases in case of HNSCC. Mainly because of a limited animal number in compliance with the 3Rs and the high inter-individual variance as described above it was difficult to achieve significant results, but a promising trend is visible. Another point that has to be mentioned is the time between drug administration in the group of combinatory treatment. Further studies have to be performed if a simultaneous drug administration is able to generate more significant results. It could be possible that tumor cells bear any additional mechanisms for better adjustment to ER-stress induction besides the already known recovER-phagy. The generation of *SEC62*-knockout clones was an important step to build a basement for further basic research in order to elucidate the molecular backgrounds underlying the clinical effects of a *SEC62* overexpression in human cancer. Here, further investigations have to be performed to investigate the sensitivity of CRISPR-Cas9 *SEC62* knockout cells regarding their migratory behaviour under TFP and TG treatment as well as under the combinatory therapy. In additional experiments different stress sensors like autophagy-dependent or apoptosis-related proteins should be investigated for upregulation or downregulation. Also, measurements of Ca^2+^ concentrations under physiological conditions in *SEC62*-knockout cells should be performed to further characterize a stable depletion of *SEC62* and its consequences for the molecular environment of a tumor cell.

Taken together, we were able to establish a reproducible new orthotopic xenograft mouse model with a metastasis induction rate of nearly 67%. Using this new model system, we investigated the anti-proliferative and anti-metastatic effects of two potential new therapeutics, TFP and TG, and were able to induce a tendency for suppressing the metastasis rate in addition to a significant reduction in metastasis size, which will be further investigated. Additionally, we were able to generate two stable *SEC62*-knockout HNSCC cell lines that were characterized and investigated for the role of SEC62 in proliferation and migration as well as the role of SEC62 in Ca^2+^ homeostasis. These cell lines will be subject of further investigations to shed light on the function of SEC62 in various processes in HNSCC cancer cell biology. With these results we are taking a first step towards a new therapeutic approach in head and neck cancer with the long-term goal of improving the prognosis of this hard-to-treat cancer entity and target the molecular mechanism of cancer metastasis.

## Data Availability

The original contributions presented in the study are publicly available. This data can be found here: http://www.ncbi.nlm.nih.gov/bioproject/809879.
