# Supplementary material for: Antagonizing Sec62 function in intracellular Ca2+ homeostasis represents a novel therapeutic strategy for head and neck cancer
Source: Front Physiol. 2022 Aug 15;13:880004. doi: 10.3389/fphys.2022.880004 (PMC9421371; doi:10.3389/fphys.2022.880004)
Supplement: Supplementary file 1 [file Table1.DOCX]

Supplementary Material

# Supplementary Figures


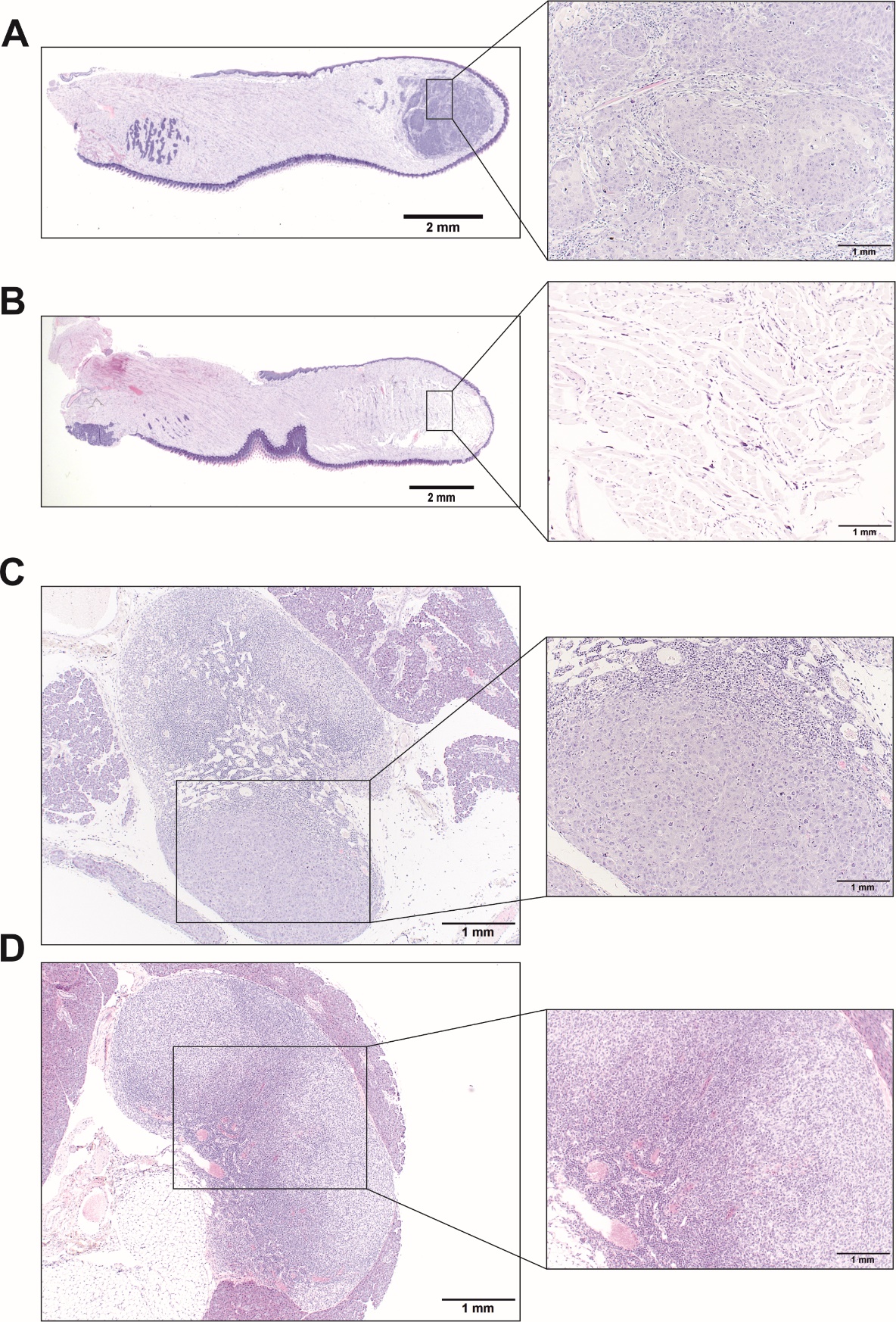


**Supplementary Figure 1.** **(A, B)** Histological section of a of a tumor-bearing tongue compared to a non-tumor bearing tongue. **(C, D)** Histological section of a lymph node containing a metastasis compared to a metastasis-free lymph node. A-D: H&E staining. A-B 1.0x; C-D 4x. A-D magnifications 10x.

#
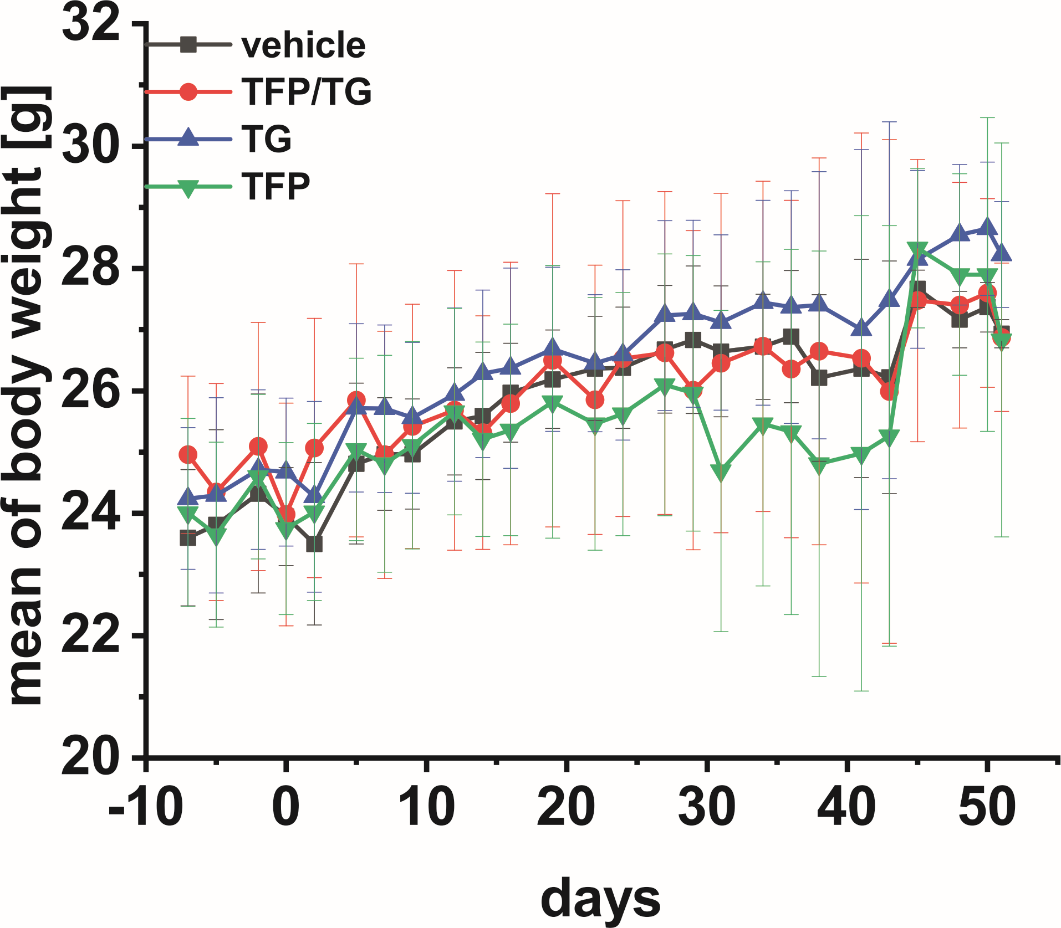


# Supplementary Figure 2. Course of the mean body weight of all injected animals categorized into their respective treatment groups with the associated standard deviation as error bars.

**Supplementary Table 1.** Raw data of the resected lymph nodes and the corresponding number of detectable metastases categorized into their respective treatment groups. LN, lymph node; Met, metastasis.

|  | **animal** | **LN sum** | **Met sum** |  | **animal** | **LN sum** | **Met sum** |  | **animal** | **LN sum** | **Met sum** |  | **animal** | **LN sum** | **Met sum** |
| --- | --- | --- | --- | --- | --- | --- | --- | --- | --- | --- | --- | --- | --- | --- | --- |
| **vehicle** | #1 | 3 | 0 | **TFP/TG** | #1 | 4 | 3 | **TFP** | #1 | 1 | 0 | **TG** | #1 | 3 | 1 |
|  | #2 | 4 | 1 |  | #2 | 4 | 0 |  | #2 | 2 | 1 |  | #2 | 4 | 1 |
|  | #3 | 3 | 1 |  | #3 | 5 | 2 |  | #3 | 4 | 2 |  | #3 | 3 | 0 |
|  | #4 | 4 | 0 |  | #4 | 5 | 2 |  | #4 | 4 | 0 |  | #4 | 2 | 0 |
|  | #5 | 6 | 5 |  | #5 | 3 | 0 |  | #5 | 4 | 0 |  | #5 | 4 | 2 |
|  | #6 | 4 | 1 |  | #6 | 5 | 2 |  | #6 | 3 | 0 |  | #6 | 4 | 1 |
|  | #7 | 5 | 3 |  | #7 | 4 | 0 |  | #7 | 4 | 0 |  | #7 | 4 | 1 |
|  | #8 | 3 | 0 |  | #8 | 4 | 1 |  | #8 | 5 | 1 |  | #8 | 6 | 4 |
|  | #9 | 5 | 4 |  | #9 | 3 | 3 |  | #9 | 5 | 2 |  | #9 | 3 | 0 |
|  |  |  |  |  | #10 | 5 | 1 |  | #10 | 4 | 3 |  | #10 | 4 | 1 |
|  |  |  |  |  | #11 | 3 | 0 |  |  |  |  |  |  |  |  |


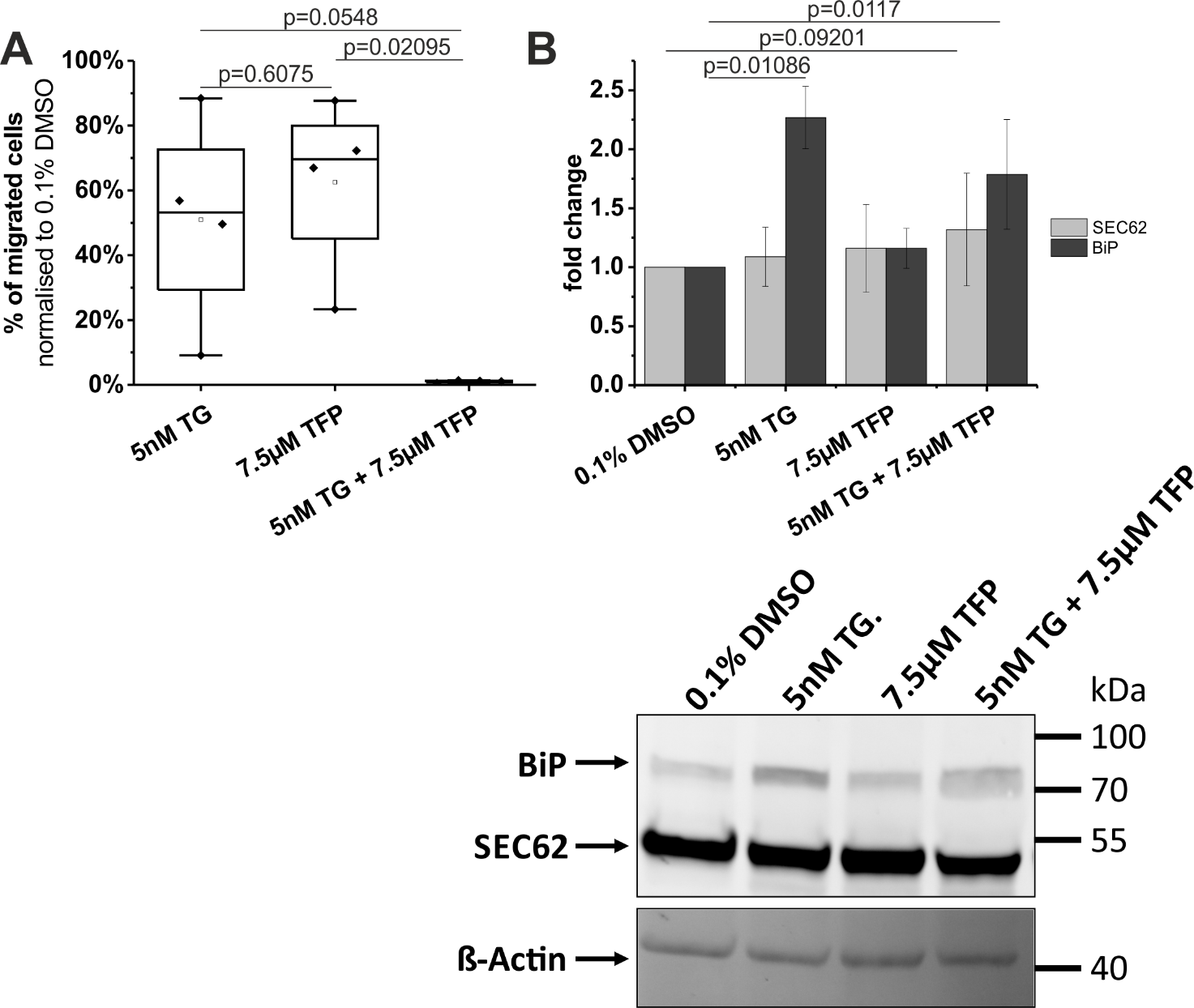
**Supplementary Figure 3.** Impact of TG and TFP treatment on FaDu wt cells. **(A)** Migratory behaviour of FaDu wt cells treated with TG and TFP. Data shows the mean of 4 biological replicates whereas 3 images were analysed in each technical duplicate. **(B)** Western blot analysis of FaDu wt cells that were treated with TG and TFP concerning BiP and SEC62 expression. Fold changes were calculated as a mean of 4 biological replicates.

**
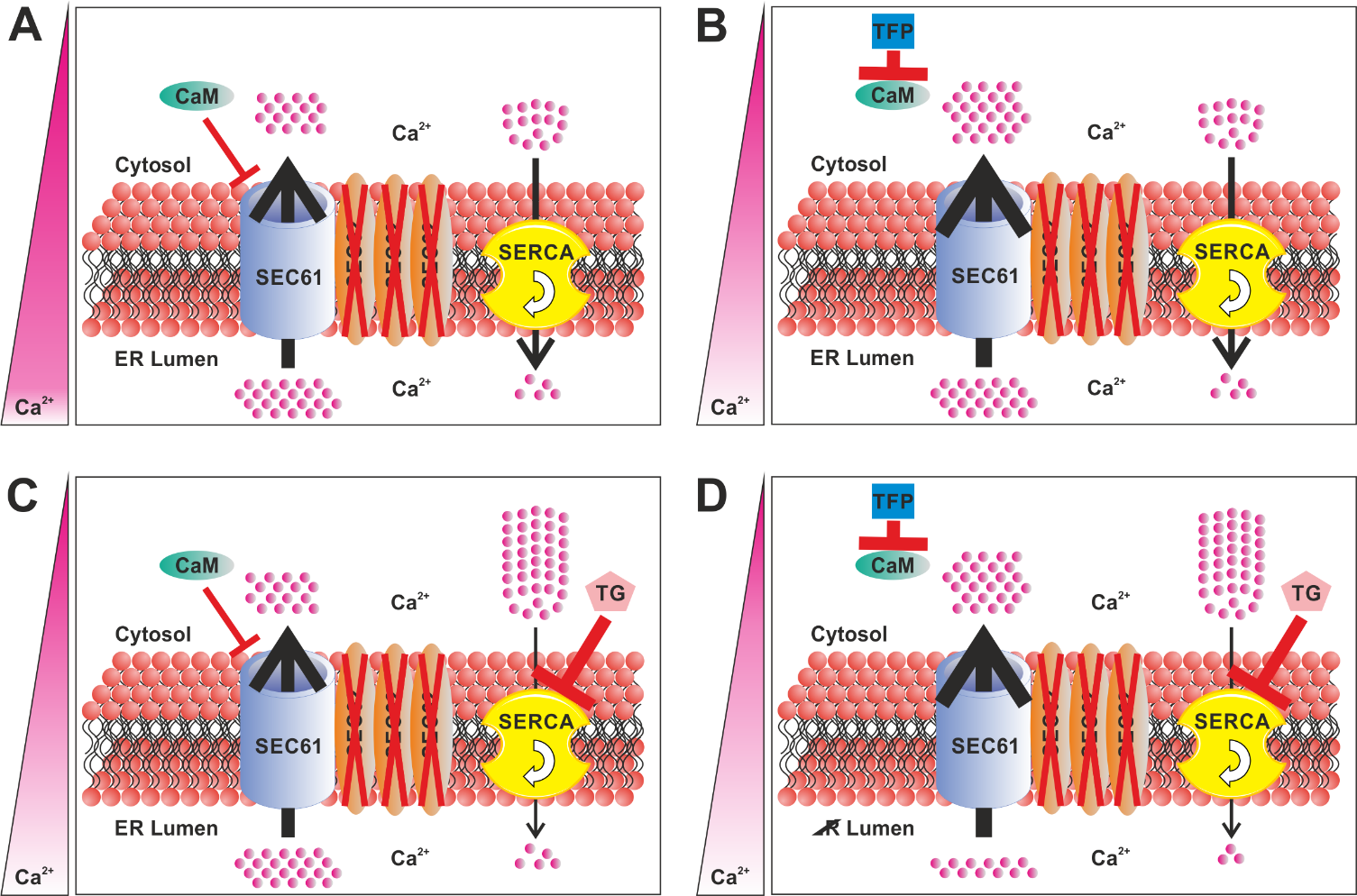
**

**Supplementary Figure 4.** The role of a *SEC62*-knockout in Ca^2+^ homeostasis of the ER in human cancer cells. **(A)** Role of a *SEC62*-knockout in the regulation of the Ca^2+^ efflux. **(B)** Differences in the Ca^2+^ efflux and resulting Ca^2+^ homeostasis under TFP treatment by the inhibition of CaM. **(C)** Differences in the Ca^2+^ efflux and resulting Ca^2+^ homeostasis under TG treatment by inhibition of the SERCA. **(D)** Differences in the Ca^2+^ efflux and resulting Ca^2+^ homeostasis under a consecutive treatment of TFP and TG by blocking CaM and through the inhibition of the SERCA. The red bars indicate an inhibition and/or channel closure. Thickness of the arrows indicates the strength of Ca^2+^efflux and the flow rate of the SERCA. The pink bar on the left indicates the Ca^2+^ balance between the ER and the cytosol. A dark pink color indicates a high Ca^2+^ level, whereas white color indicates a low Ca^2+^ level. CaM, calmodulin; TFP, trifluoperazine; TG, thapsigargin; SERCA, sarcoplasmic/endoplasmic reticulum Ca^2+^-ATPase.
